# Recess and after-hours behavior patterns associated with community schoolyard transformations

**DOI:** 10.1186/s12966-026-01899-9

**Published:** 2026-03-13

**Authors:** Marnie F. Hazlehurst, Kathleen L. Wolf, Cary Simmons, Sarneshea Evans, Mary Kathleen Steiner, Kimberly A. Garrett, Pooja S. Tandon

**Affiliations:** 1 Seattle Children’s Research Institute, Seattle, WA USA; 2 University of Washington, Seattle, WA USA; 3 Trust for Public Land, Washington, DC USA; 4 Department of Pediatrics, University of Washington, Seattle, WA USA

**Keywords:** Schools, Physical activity, Shared use, Green schoolyards

## Abstract

**Background:**

Spending time in nature and physical activity are both linked to better health outcomes for children. School environments provide the potential for multiple interventions to promote healthy behaviors. Transforming these existing public spaces to serve broader activity functions provides opportunities to support healthy child development and to increase nature access for local communities.

**Methods:**

Our quasi-experimental design capitalized on a community-engaged schoolyard redesign and redevelopment project completed at two elementary schools in Tacoma, Washington, USA, led by the Trust for Public Land. Renovations included painting paved surfaces, installation of new play structures, adding walking paths and log seating areas, and planting trees. We used a validated momentary time sampling observational tool, the System for Observing Outdoor Play Environments in Neighborhood schools (SOOPEN), before and after for two schools undergoing the transformation and a control school. Moderate-to-vigorous physical activity (MVPA) and within-group behaviors in the schoolyard were observed. We used a difference-in-difference approach to estimate the effect of the schoolyard renovation on behavior during recess, and on community use of the schoolyard outside of school hours.

**Results:**

The schoolyard transformation was associated with a 1.51-times greater increase (95% CI: 0.95, 2.40) in MVPA during recess at intervention schools compared to the control school, although this association was only statistically significant among groups of boys. After renovation, MVPA was highest in grassy areas at one school (69%), but highest in paved zones with newly painted markings at the other (75%). Renovations were not associated with changes in the prevalence of prosocial behavior during recess, which was high at baseline. The schoolyard transformation was also associated with increased community use outside of school hours, especially for groups of children (5.8-times [95% CI: 1.4, 23.8] greater increase in use over time at renovation schools compared to the control school).

**Conclusions:**

Schoolyard transformations have the potential to increase schoolyard use and physical activity both during and outside of school hours, if campuses are open to community use. Additional rigorous research can inform future projects to support the health of school-age children and address inequities in distribution of community greenspaces.

**Supplementary Information:**

The online version contains supplementary material available at 10.1186/s12966-026-01899-9.

## Background

Lack of physical activity and poor mental health in childhood have been linked to worse health outcomes across multiple domains throughout childhood and into adulthood, making child physical activity a key outcome of critical public health importance [1, 2]. However, many children are not achieving the recommended levels of daily activity [3], and multi-level solutions are needed [4]. Lower rates of physical activity have been identified for children with lower socioeconomic status, those living in areas lacking access to greenspace or safe outdoor spaces for children to play, and among girls [5, 6].

In addition, spending time in nature has been linked with better health outcomes across multiple domains. Access to greenspace, including both public parks and overall greenness surrounding the home, is associated with a variety of health outcomes in children, including reduced behavioral problems [7, 8] and increased physical activity [9]. School environments potentially augment the benefits of community greenspace as school-age children spend a large portion of their time at school and attain a large percentage of their daily physical activity at school [10]. School recess provides important opportunities for free play, physical activity, motor skill and social and emotional development [11].

Increased recognition of potential health benefits to children has led to efforts to improve access to green spaces, as well as the quality of existing green spaces. However, in many communities the creation of new park spaces is not feasible due to limited land availability, high real estate costs and stretched public budgets. School environments are potential locations for multiple interventions to promote healthy behaviors and development [12, 13]. Thus, transforming existing public spaces, such as schoolyards, provides opportunities to increase nearby nature access and promote children’s health.

Research is limited concerning the relationship between physical schoolyard conditions and children’s physical activity behavior. A recent review including studies of children and adolescents ages 0–17 yrs, found that while playground markings in schoolyards were associated with greater physical activity, greening did not necessarily have the same clear relationship with physical activity [14]. Renovation programs provide analysis opportunities. Studies of interventions focused primarily on greening schoolyards have not consistently demonstrated an association with higher physical activity, though some do suggest benefits for both physical activity and prosocial behavior [14, 15]. Studies of a small number of renovated elementary schoolyards in Chicago, IL identified lower rates of sedentary activity or higher rates of MVPA after, compared to before the renovation, but lacked a control location [16, 17]. Increases in prosocial behavior or positive interactions in schoolyards after, compared to before renovation, were also identified [16]. Comparisons of nine elementary schools in Denver, CO found higher overall levels of physical activity in renovated schoolyards [18], but only among boys when comparing the proportion of students engaging in MVPA [19]. A study in Cleveland, OH found a difference in MVPA between renovated and control elementary schoolyards only among boys, as well as higher schoolyard utilization overall outside of school hours, at the renovated schoolyards [20]. The effects of any intervention may vary across subgroups as well; comparisons have identified weaker relationships between schoolyard renovations and physical activity among the least-active children [21].

We note that evaluation of schoolyard greening efforts has frequently been limited by small sample sizes, study design, and potential bias from confounding factors. Few prior studies have utilized both pre and post intervention data, or a control group at both time points. Two such studies found that greening schoolyards increased physical activity among girls and improved some behavioral or attention-related outcomes [22, 23]. One of these studies was conducted in The Netherlands in 2014–2016 and examined changes in MVPA in five intervention and four control schoolyards [22]. Physical activity during recess, assessed via accelerometry, was associated with schoolyard greening, but only among girls. The study also found that greening improved performance on attention tests and social well-being, but not emotional well-being. Another study was conducted at two schools in Los Angeles, CA and similarly found an association of schoolyard greening with increased physical activity only for girls [23], in addition to decreased antisocial behavior across genders. A study in the London Borough of Camden included five intervention and two control schools, and utilized accelerometry to assess student physical activity [24]. Schoolyard renovations included play structures and artificial turf areas, designed in consultation with children and teachers at each school. No overall changes in overall physical activity were detected after renovations, yet there were associations with decreased sedentary activity and increased light-intensity physical activity among children under nine years of age.

Our study utilized a quasi-experimental design to capitalize on schoolyard transformations completed in Tacoma, Washington, U.S.A. These school-based projects are part of a national program sponsored by the Trust for Public Land to transform schoolyards into community greenspace and involve students and community members in the design process [25]. The first aim of this study was to examine whether the physical facilities changes were associated with child physical activity or prosocial behavior during recess, and differences by gender, using data collected before and after for two schools undergoing interventions and a control school. Our second aim was to explore whether child behavior varied by schoolyard area type, including newly built nature areas, at renovated schoolyards. In addition, we aimed to assess user density and attributes of visiting community members outside of school hours, including multi-generational groups.

## Methods

### Community schoolyard campaign

This study was designed to evaluate outcomes of community schoolyard transformations in Tacoma, WA, U.S.A., conducted by the Trust for Public Land (TPL) as part of the national Community Schoolyard Initiative. TPL is partnering in communities to expand park access for millions of people across the United States by transforming schoolyards and facilitating shared use agreements especially in areas with inequitable park access [26]. Planned schoolyard conversions in Tacoma will serve more than 40,000 people who currently do not have a park near their home. The entire cohort of six elementary schools selected for renovations primarily serve children ages 5–10 in kindergarten to grade 5, with some younger students in pre-kindergarten Head Start programs at the schools. All are located within the southeast area of Tacoma, are socio-economically diverse (more than 70% of families at all three schools identified as low income during the 2022-23 school year), have low levels of opportunity based on the Tacoma Equity Index Map, and exhibit some of the poorest public health metrics across the city [25]. TPL facilitates a community-engaged process with each participating school, resulting in substantial re-design, leads fund-raising efforts for renovation costs, and supports the shared use process. Two schools were early participants and construction has been completed; a third school (slated for later renovation) served as a control based on similar geographic and demographic factors. Shared use agreements were established between Tacoma Public Schools and Parks Tacoma at the time of renovation.

Both the Seattle Children’s Hospital (STUDY00002677) and University of Washington (STUDY00011253) Institutional Review Boards approved this study and determined it to be exempt. Tacoma Public Schools’ Data and Assessment Research Team (DART) and leadership at each school agreed to data collection on their campus by study staff.

### Site renovations

**Fig. 1:**
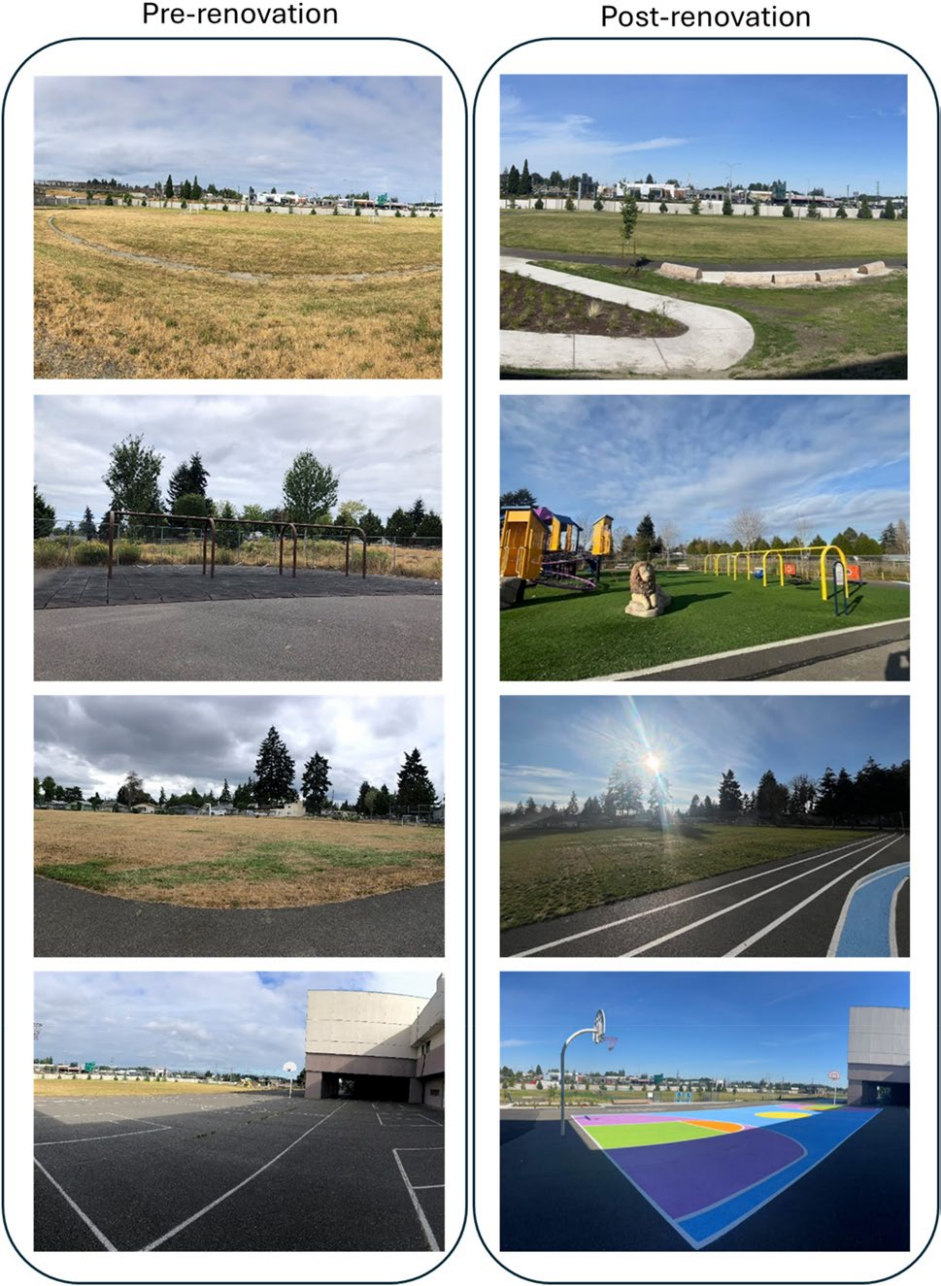
Schoolyards before and after community engaged redesign and renovation. Schoolyard renovations were designed with extensive student and community input and included installation of new play structures; painting paved surfaces with bright, cheerful colors; improvements to existing grassy fields including improving drainage of surfaces during rainy winter months; adding a paved walking path around a sports field and a paved access path; adding outdoor gathering/learning space including logs arranged as seating areas; and creating small nature areas, including planting trees and other vegetation

A participatory design process engaged students (third graders initially and then the entire school) in ideation and visualization of the future schoolyard. Philanthropic fundraising, supplemented with agency funding from Parks Tacoma and Washington State Office of Recreation and Conservation, provided fiscal support, with final renovation managed by professional design and construction firms. The components of TPL schoolyard transformations vary by geography. In Tacoma renovations included removal of old play structures and installation of new play structures; painting existing paved surfaces with basketball court lines, foursquare courts, and patterns of bright, cheerful colors; improvements to existing grass-covered fields; adding a paved walking path around a sports field and a paved access path; adding outdoor gathering/learning space (e.g. log seating areas); and creating small nature areas, including planting trees and other vegetation (Fig. 1). Renovations were completed and schoolyards re-opened for student and community use in the first half of 2024.

### Schoolyard observations

To assess play behavior and social interactions in the schoolyards, we utilized the System for Observing Outdoor Play Environments in Neighborhood schools (SOOPEN) [27, 28]. SOOPEN is a tool using momentary time sampling for observations of play settings to assess physical activity and social behavior at the group level and demonstrates good inter-rater reliability [29]. SOOPEN uses group-level observations to better record within-group dynamics that may not be captured by individual-level observation tools, thus acknowledging the interdependent relationships of social interactions and social networks with physical activity in the context of outdoor play.

Prior to schoolyard renovations, data were collected using SOOPEN during the spring and summer of 2022 at both intervention schools and the control school. SOOPEN was implemented again in June 2024 at all three schools, to collect data after construction completion. As previously described [29], each schoolyard was divided into target areas based on defined boundaries within and around the schoolyard. Target areas were then scanned by trained observers. Target areas were categorized based on the primary features of the schoolyard area: field or grassy area, paved surface, or play structure. Target area boundaries generally remained the same pre- to post-renovation, except when play structures were moved to a different area of the schoolyard. While both schools had an added nature area, as well as natural elements integrated throughout the schoolyard, one school added a distinct nature area, featuring logs for seating and new plantings, that was treated as a separate target area.

Observation sessions were conducted during lunch recess, afterschool, evenings, and weekends. Recess, afterschool, and evening observations were conducted on two days at each school pre-renovation in May 2022 and on two days at each school post-renovation in June 2024. All recess and afterschool observations were conducted during the school year while school was in session. Weekend sessions were scheduled during four time windows (morning, early afternoon, late afternoon, and evening) on two weekend days at each school pre-renovation in May 2022 (during school year) and August 2022 (during summer break) and on two weekend days post-renovation in June 2024 (one during school year and one during summer break).

During each session, observers scanned the entirety of the target zone from a predetermined position and recorded information about each group observed within the scan. Group size (small [2–4 individuals], medium [5–9 individuals], large [10 or more individuals], or an individual alone), perceived gender of the group (only girls, only boys, or mixed-gender group), and age group (children, teens, adults, seniors, or age-mixed) were recorded for each group. Groups including children or teens as well as adults or seniors were categorized as multigenerational. Information on group physical activity and social behavior were also recorded, as described below.

#### Non-recess schoolyard use

Use of the schoolyard after school and during evenings and weekends was assessed as the number of groups observed within a specific target area during a given scan.

#### Physical activity

Observers recorded activity using five categories: lying down (1), sitting (2), standing (3), walking (4), or vigorous (5). Activities coded as vigorous [5] included running, hopping, or other fast movements within or through the target area. Categories 1, 2, and 3 were combined during analysis as sedentary for descriptive purposes. For regression analyses, we dichotomized the scale as category 5 compared to categories 1–4 to define the outcome of moderate-to-vigorous physical activity (MVPA). This cutpoint between walking and vigorous categories has been validated with accelerometry as an appropriate measure of MVPA in children [30]. As a secondary measure, we examined MVPA or walking as a composite outcome, defined as categories 4 or 5. Outdoor or natural environments may facilitate movements that occur while sitting or standing, including to improve balance, flexibility and build muscle strength; however, our study focused on MVPA due to links between higher intensity activity and health outcomes.

#### Prosocial behavior

Social interactions were recorded for each observed group in five categories: neutral, physical pro-social behaviors, non-physical pro-social behaviors, physical conflict, and non-physical conflict. To generate a binary prosocial behavior measure, physical and non-physical prosocial behaviors were combined as prosocial behavior, and all other behaviors were coded as not prosocial. Physical pro-social behaviors include playing a team or group game together, assisting others to stand or move, pushing others on swings, walking or running together, fetching equipment for others, and holding hands/linking arms. Verbal pro-social behaviors include saying thank you, praising others, and providing rules or instructions to organize a game.

### Statistical analysis

We examined the number and percent of groups during recess by physical activity level, prosocial behavior, group size, and perceived gender, observed at intervention and control schools, at baseline and at post-renovation follow-up. In this quasi-experimental study design, we used a difference-in-differences (DID) approach to assess the effect of schoolyard renovations on physical activity and prosocial behavior during recess. We modeled group-level physical activity and prosocial behavior using a Poisson regression model with robust standard errors. We further explored differences in physical activity over time in separate models for the intervention schools and the control school. These analyses were all conducted at the group level. We report effect estimates and 95% confidence intervals (CI) for each analysis, as well as p-values. Our interpretation acknowledges trends and effect sizes in addition to statistical significance, given that our study is contributing to an emergent literature on schoolyard conditions and children’s physical activity. This approach to reporting may aid future schoolyard designers and researchers to further specify and evaluate physical schoolyard interventions.

For the second aim of the study, we examined MVPA at intervention schools at post-renovation by group location—field/grassy area, area with paved surface such as basketball courts, play structure areas, and the newly added nature area—using Poisson regression with robust standard errors in a model adjusted for school. In a post-hoc exploratory analysis we also examined separate models stratified by school.

We also described schoolyard use outside of school hours (afterschool, evenings, and weekends) as the number of groups observed per scan, as well as by age group (child-only groups versus multigenerational groups), proportion of groups in MVPA during a scan, and proportion of groups exhibiting prosocial behavior during a scan. We used a DID approach to estimate the effect of the schoolyard renovation on community use of the schoolyard outside of school hours, on both a relative scale using Poisson regression and an absolute scale using linear regression with robust standard errors. We also examined schoolyard use over time at intervention schools and the control school separately. All analyses were conducted in R 4.4 [31].

## Results

### Recess

During recess, a total of 1479 groups were observed. Table 1. shows distribution of groups by intervention schools versus control school, and by observation time pre- versus post-renovation. The percent of groups engaged in MVPA ranged from 18% to 26%. The proportion of groups engaged in conflict behavior was low across all locations and times (< 3%), while the proportion engaged in prosocial behavior was greater than 50%. Across all observation timepoints, all-girls groups were most common, followed by all-boys groups, with mixed-gender groups being least common. Children were most frequently observed in small grouat the control school, we found that the increaseps or alone.

**Table 1:** Characteristics of groups observed using SOOPEN during recess

	**Intervention**		**Control**	
	**Pre**	**Post**	**Pre**	**Post**
Number of groups	N=676	N=490	N=157	N=156
Activity level				
Sedentary	34%	16%	40%	31%
Walking	48%	61%	34%	47%
Moderate/Vigorous	18%	23%	26%	22%
Social interactions				
Prosocial – physical	21%	23%	28%	27%
Prosocial – verbal	34%	38%	36%	46%
Neutral	42%	39%	34%	28%
Physical conflict	2%	0%	1%	0%
Verbal conflict	1%	<1%	1%	0%
Perceived gender				
Girls only	43%	41%	38%	44%
Boys only	34%	36%	35%	40%
Mixed group	23%	22%	27%	17%
Group size				
Alone	40%	38%	30%	29%
Small	51%	51%	50%	59%
Medium	7%	10%	14%	10%
Large	3%	2%	6%	2%
Group location				
Field/grass	33%	29%	16%	26%
Paved	35%	34%	42%	38%
Play structure	31%	27%	42%	36%
Nature area	-	10%	-	-

At schools that underwent a schoolyard renovation, we found a 30% increase (RR 1.30, 95% CI: 1.04, 1.64) increase in the probability of a group in the schoolyard engaging in MVPA over time from pre- to post-renovation (Supplemental Table S2). In stratified analyses by gender (Supplemental Table S2), similar trends were observed among single-gender groups, but not mixed-gender groups (RR 0.92, 95% CI: 0.60, 1.42, *p* = 0.784), though estimates were statistically significant only among boys (RR 1.68, 95% CI: 1.10, 2.54, *p* = 0.016) and not girls (RR 1.38, 95% CI: 0.95, 2.00, *p* = 0.109). In contrast, we estimated a 14% decrease in MVPA over time (RR 0.86, 95% CI: 0.57, 1.28, *p* = 0.503) at the control school, though not statistically significant (Supplemental Table S2).

When the change over time at the intervention schools was compared to the change over time at the control school, we found that the increase in groups engaging in MVPA during recess was 1.51-fold higher (95% CI: 0.95, 2.40, *p* = 0.080) at intervention schools compared to the control school (Table 2). This trend was observed in single-gender but not mixed-gender groups and was statistically significant among groups of boys only (difference-in-difference relative risk ratio [DID RRR] 2.13, 95% CI: 1.03, 4.41, *p* = 0.041).

**Table 2:** Estimated effect of schoolyard renovation on moderate-to-vigorous physical activity (MVPA) and prosocial behavior during recess

Outcome and sample	DID RRR estimate (95% CI)	*p*-value
MVPA		
All groups^a^	1.51 (0.95, 2.40)	0.080
Groups of girls	1.79 (0.82, 3.93)	0.145
Groups of boys	2.13 (1.03, 4.41)	0.041
Mixed gender groups	0.73 (0.28, 1.91)	0.558
MVPA + walking		
All groups	1.12 (0.94, 1.34)	0.237
Prosocial behavior		
All groups	0.96 (0.80, 1.15)	0.683

*Abbreviations:* *DID RRR* Difference-in-difference relative risk ratio, *MVPA* Moderate-to-vigorous physical activity

^a^All groups includes the entire sample: single gender groups (girls-only and boys-only) as well as mixed-gender groups

We examined the use of renovated schoolyards by target area after interventions (Table 3). When both schools were combined in a single model adjusted for school as a fixed effect, we found that play structure areas were associated with less MVPA compared to grassy areas (PR 0.24, 95% CI: 0.13, 0.45, *p* <0.001) and other relationships with MVPA or prosocial behavior were not statistically significant. However, in models stratified by school we found distinct trends at each school. At the larger of the two renovated schoolyards, paved areas were associated with less MVPA compared to grassy areas (PR 0.37, 95% CI: 0.18, 0.76, *p* =0.014) and play structure areas were associated with less MVPA compared to grassy areas (PR 0.15, 95% CI: 0.05, 0.46, *p* =0.001). At this school, there were five target areas designated as field/grass or nature areas and these areas were both frequently used and had a high prevalence of MVPA. In contrast, no significant associations between target area type and MVPA were observed at the smaller schoolyard. In contrast to the larger schoolyard, the direction of the effect estimate for the smaller schoolyard suggests more MVPA in paved areas versus grassy areas (PR 2.73, 95% CI: 0.88, 8.49, p=0.098). Paved areas were also associated with less prosocial behavior than grassy areas at the smaller schoolyard. Differences between the structure of the two intervention schoolyards and the delineation of target areas at each schoolyard may contribute to some of these differences. The field at the smaller schoolyard was considered as a single target area, the nature area was included as part of the field target area, and nature features were added to multiple zones.

**Table 3:** MVPA and prosocial behavior during recess in transformed schoolyards

Group location	Combined (*N* = 491)		School 1^a^ (*N* = 242)		School 2 ^a^ (*N* = 249)	
	PR (95% CI)	*p*-value	PR (95% CI)	*p*-value	PR (95% CI)	*p*-value
MVPA						
Field/grassy area	*1 (reference)*		*1 (reference)*		*1 (reference)*	
Paved area	0.73 (0.51, 1.06)	0.200	0.37 (0.18, 0.76)	0.014	2.73 (0.88, 8.49)	0.098
Nature area	0.69 (0.41, 1.15)	0.234	0.60 (0.36, 1.00)	0.104	--	--
Play structure	0.24 (0.13, 0.45)	< 0.001	0.15 (0.05, 0.46)	0.001	0.91 (0.25, 3.34)	0.888
Prosocial behavior						
Field/grassy area	*1 (reference)*		*1 (reference)*		*1 (reference)*	
Paved area	0.73 (0.60, 0.90)	0.063	0.99 (0.74, 1.34)	0.958	0.56 (0.44, 0.70)	0.009
Nature area	0.92 (0.69, 1.21)	0.693	1.01 (0.75, 1.38)	0.979	--	--
Play structure	1.04 (0.88, 1.24)	0.785	1.14 (0.87, 1.49)	0.558	0.86 (0.71, 1.03)	0.479

*Abbreviations:* *PR* Prevalence ratio

^a^School 1 (11 target areas) was larger than school 2 (5 target areas)

### Community use of schoolyards

Outside of school hours, a total of 494 scans were conducted (79 during afternoons directly after school, 79 in the evening, and 336 on weekends) and 280 groups observed (Table 4).

**Table 4:** Characteristics of SOOPEN observations during afterschool, evening, and weekend hours

Scan characteristic	Intervention		Control	
	Pre	Post	Pre	Post
Total number of scans (n)	216	182	48	48
Number of groups observed (n)	82	102	66	30
Percent of scans with 1 or more groups observed (%)	15.7	25.8	33.3	31.2
Number of groups per scan, mean (SD)				
All groups	0.38 (1.10)	0.56 (1.43)	1.38 (3.39)	0.62 (1.21)
Child-only groups	0.17 (0.69)	0.25 (0.82)	0.90 (2.43)	0.23 (0.78)
Multi-generational groups	0.13 (0.48)	0.15 (0.43)	0.19 (0.45)	0.23 (0.59)
Proportion of groups in MVPA, mean (SD)	9.1 (23.8)	14.0 (29.6)	16.6 (27.4)	17.8 (37.5)
Proportion of groups with prosocial behavior, mean (SD)	61.0 (41.8)	52.7 (44.7)	55.9 (40.5)	64.4 (44.5)

Decreases in all groups and child-only groups were observed over time at the control school, whereas increases in schoolyard use were observed over time at intervention schools, though these changes were not generally statistically significant (Supplemental Table S3). For example, use of the schoolyard at intervention schools increased by 1.48-times (95% CI: 0.72, 3.02, *p* =0.153). Using a difference-in-difference approach, we estimated the effect of the schoolyard renovation on both relative and absolute scales (Table 5). Schoolyard renovation was associated with a 3.2-fold greater (95% CI: 1.2, 9.1, *p* =0.025) increase in use of the schoolyard by the community outside of school hours at intervention schools compared to control schools. When stratified by age group, this increase was observed for groups of children, but estimates for multi-generational groups were not statistically significant. Similar trends were observed on the absolute scale using the difference-in-difference absolute difference estimate (DID AD).

**Table 5:** Estimated effect of schoolyard renovation on community use of the schoolyard

	*N* of scans	*N* of groups observed	DID RRR estimate (95% CI)	*p*-value	DID AD estimate (95% CI)	*p*-value
All groups	494	280	3.2 (1.2, 9.1)	0.025	0.93 (−0.11, 1.97)	0.080
Child-only groups	494	137	5.8 (1.4, 23.8)	0.015	0.75 (0.01, 1.48)	0.046
Multi-generational groups	494	76	0.9 (0.28, 2.92)	0.866	−0.03 (−0.25, 0.20)	0.811

*Abbreviations:* *DID RRR* Difference-in-difference on the relative risk ratio scale, *DID AD* Difference-in-difference on the absolute difference scale

## Discussion

This study examined two schoolyards that were transformed with student and community input, and a control schoolyard, at elementary schools (grades K-5) in Tacoma, WA using the SOOPEN observational tool. An important finding of our study was that the schoolyard intervention was associated with increased community use outside of school hours, especially for groups of children. We also found that schoolyard renovation was associated with increased probability of groups engaging in MVPA during recess at intervention schools compared to the control school over time in all groups, though this finding was statistically significant only for groups of boys. After renovation, the target areas with the highest prevalence of MVPA varied by school, with grassy areas having the most MVPA at one school and paved areas with bright, newly painted markings at the other school. Renovations were not associated with changes in prevalence of prosocial behavior, which was high at baseline.

Many of the prior studies of renovations to improve schoolyard facilities have either (1) compared intervention and control schoolyards without data from before the renovation or (2) compared schoolyard activity before and after renovation but without a control school. As has been identified in prior reviews [14], we observe that only a small number of prior studies on this topic have similarly used controls at both pre- and post-renovation timepoints [22–24]. Importantly, our study improves upon much of the prior research by utilizing a control school at both time points to robustly control for confounding in the study design.

Our study adds to existing evidence of schoolyard greening and/or comprehensive schoolyard transformations increasing physical activity among children [16, 17, 21–23]. Two studies, one in The Netherlands and one in the US, using a similar study design to ours, found statistically significant increases in physical activity only among girls [22, 23], while others observed changes in light physical activity only for younger children (< age 9) [24] or only among the least-active children at baseline [21], in England and Denmark, respectively. In contrast to these prior studies, our study identified an association between the schoolyard renovation and MVPA that was only statistically significant for groups of boys. The estimate across all groups and for groups of girls were both of a similar magnitude to that among boys; however, estimates for all groups and for groups of girls did not reach statistical significance. Differences from prior work may be a result of our measurement of physical activity at the group level, using a tool that captures social dynamics of single-gender versus mixed gender groups, or due to other local cultural or geographical differences in the samples. In addition, the specific features and amenities of each schoolyard differed initially and as a result of independent participatory design processes, so likely support physical activity of subgroups differentially, making it challenging to compare across schools. Future work should consider the effect of the intervention within subgroups with lower baseline levels of physical activity.

A key finding of our study was the increase in use of the transformed schoolyards by the community outside of school hours. In addition to promoting healthy behaviors during recess, a primary goal of the Trust for Public Land’s program of schoolyard transformations is to provide a community resource in neighborhoods lacking access to parks and other greenspaces. Community use of the schoolyards in our study was limited during evenings and weekends at baseline [29]. However, we found that renovations were associated with a notable increase in community use of the schoolyard outside of school hours, including almost 6 times greater increase in use over time by children at intervention compared to control schools. This finding is consistent with prior studies that identified increased utilization of parks or schoolyards after renovations, irrespective of whether the renovation was associated with increased physical activity [20, 32–34]. Others have hypothesized that an increased sense of safety in renovated schoolyards may facilitate increased community use [16, 32], as perceived safety of the schoolyard is associated with use of these spaces by community members [35]. The increase in use and physical activity observed in our study after renovations in two schoolyards is a compelling finding in support of green and community-oriented schoolyards as a strategy for increasing public access to greenspaces. This study also found that most of the outside-of-school hours use was by groups of children. While we do not know if these were children that currently or previously attended the school or live nearby, it is encouraging that schoolyard renovation and after hours shared use is facilitating active, outdoor recreation for children and access to spaces to engage in health promoting behaviors. Additional work needs to focus on how to further activate open schoolyards by implementing amenities, programming, and community engagement, including strategies targeting multi-generational use. Given the disparities of park access in many communities, often associated with sociodemographics, schoolyards are a ubiquitous public space resource in the U.S. Continued research, based on our evidence, is important for informing advocacy, policies, and investment to promote public health for people of all ages.

With regard to existing evidence of benefits of nature contact for child mental health [9] and the potential pathways of social cohesion by which greenspace may facilitate positive health outcomes, several studies have explored the effect of schoolyard renovations on child prosocial versus antisocial behavior and social well-being [16, 22, 23]. There are prior identified increases in prosocial behavior, positive interactions, and social-wellbeing or decreases in antisocial behavior, in post-renovation schoolyards. In contrast to these earlier studies, we did not find any associations with prosocial behavior during recess. However, high baseline levels of prosocial behaviors and low levels of conflict behaviors in our study, as well as occasional difficulty for observers in assessing verbal behaviors from a distance in larger or noisier target areas, may have limited our ability to detect any differences. Despite robust study designs, comparison across these studies is further complicated by the variability in the characteristics of the schoolyard renovations across studies. Furthermore, the extent of social-emotional curricula presented in schools was not accounted for in our study.

In prior studies, some renovation designs have focused on greening the schoolyard, while others have more broadly addressed schoolyard renewal or schoolyard activation for physical activity. Renovations beyond schoolyard greening include changes to play structures and modifications to paved areas, often with community input, similar to the TPL schoolyard transformations in the current study. Our study, and prior research, suggests that multiple design features may influence child physical activity and should be considered in schoolyard redesign. Of particular interest is to understand the specific spatial features and relationships of schoolyard sub-spaces that encourage physical activity and prosocial behavior. Within a schoolyard, some areas and features may facilitate active play more than others and this may differ by user age, gender, ability or other characteristics. In our study the field and grassy areas were associated with more MVPA compared to other areas of the schoolyard at one location, while paved areas with painted markings were associated with more MVPA compared to grassy areas at the other schoolyard. These results diverged from our findings pre-renovation, when MVPA was more prevalent in areas of the schoolyard with paved surfaces [29]. Our study adds to the mixed literature. Adding playground markings has consistently been associated with greater MVPA in other studies [14], though other studies have found greater MVPA in grassy areas of the schoolyard [36] or in areas with tree-provided shade and/or balance and climbing obstacles [37] and greater schoolyard use with the presence of multiple play structures and shade [38]. Furthermore, specific features promoting physical activity have been found to vary by student gender [39]. Affordance Theory offers a framework for interpretation of this literature [40, 41]; different types of outdoor spaces afford a broader set of opportunities depending on the configuration and perceived resources within the space. Schoolyard transformations may clarify the affordances of spatial areas through changes in built form and surface colors. Our findings suggest the importance of incorporating both greening elements and other design features that promote physical activity with consideration of different subgroups that may use this space. Qualitative research to explore user perceptions may further our understanding of the user responses to specific changes in spatial conditions made as part of schoolyard transformations. 

The role of weather and climate is not consistently addressed in prior research. Some studies have primarily focused on schoolyard greening as a strategy to address high temperatures. In western Washington State, the weather-related focus of the renovations included creating spaces conducive to active play during rainy, wet conditions. Renovations at both schoolyards included drainage improvements to the grass fields, as well as the installation of artificial turf in play structure areas. Future studies should consider how different surfaces are associated with physical activity including the impact of weather/climate. The variability in findings across intervention schoolyards highlights that schoolyard transformations should consider local conditions (e.g. climate), school-specific factors (e.g. schoolyard size), and community input regarding user needs.

A critical challenge in the timing of this study was the COVID pandemic, which interrupted community planning and delayed implementation of the schoolyard re-design. An additional limitation in this study is the inclusion of a relatively small sample, that being two renovated schoolyards of difference sizes and having different design features, and a single control school. Future work at a greater number of schools in different geographies, including control schools, will be important to improve generalizability of our findings. The TPL Community Schoolyard transformations assessed in our study are part of an ongoing national program, providing the potential to expand this work to larger sample sizes, across seasons and geographies, and with varying sociodemographics. Interpretation of future analyses would be further strengthened by sampling across multiple time points prior to the renovation, to test the assumption of the difference-in-differences approach that the control school provides a good proxy for the counterfactual. Lastly, we were unable to examine trends by age group or grade during recess as there were often classrooms from multiple grades using the schoolyard simultaneously, which limited our ability to draw conclusions about younger versus older children.

This work contributes to a growing body of evidence focused on physical activity and built environments [42]. Physical activity is now widely recognized as a critical determinant of physical and mental health [43], with early-life activity linked to long-term habits and reduced morbidity [44]. Outdoor activity in natural environments provides additional benefits beyond those gained indoors [45]. Yet, structural inequities persist with lower-income communities and communities of color disproportionately lacking access to safe, high-quality green spaces [46], limiting opportunities for individual and community physical activity and other health promoting behaviors.

## Conclusions

Schoolyard transformations in two schools in our study were associated with increased physical activity among boys and usage of the space after hours as a community park. Findings of similar magnitude that did not reach statistical significance among groups of girls and in the overall sample warrant further study in a larger number of schools. Our findings reinforce the value of renovating and opening schoolyards as a public health intervention to address inadequate park access while improving the daily experience of those attending the school. Investing in schoolyard transformations with community engagement can serve as a scalable and sustainable strategy across the U.S. to promote physical activity and social connection, while acknowledging that schoolyard redesigns will likely differ in form and appearance across communities. Our study contributes actionable evidence to support such initiatives, inform future research, and underscores the potential of schoolyards to become vital components of a more equitable and health-promoting built environment.

## Supplementary Information

Supplementary Material 1.

## Data Availability

The datasets used and/or analyzed during the current study are available from the corresponding author on reasonable request.

## Abbreviations

SOOPEN: System for Observing Outdoor Play Environments in Neighborhood schools
MVPA: Moderate-to-vigorous physical activity
TPL: Trust for Public Land
DART: Data and Assessment Research Team
DID: Difference-in-difference
CI: Confidence interval
DID RRR: Difference-in-difference relative risk ratio
PR: Prevalence ratio
SD: Standard deviation
DID AD: Difference-in-difference on the absolute difference scale
