# Supplementary material for: Recess and after-hours behavior patterns associated with community schoolyard transformations
Source: Int J Behav Nutr Phys Act. 2026 Mar 13;23:25. doi: 10.1186/s12966-026-01899-9 (PMC13001272; doi:10.1186/s12966-026-01899-9)
Supplement: Supplementary file 1 — Supplementary Material 1. [file 12966_2026_1899_MOESM1_ESM.docx]

**Supplemental Material**

**Recess and after-hours behavior patterns associated with community schoolyard transformations**

Marnie F. Hazlehurst, Kathleen L. Wolf, Cary Simmons, Sarneshea Evans, Mary Kathleen Steiner, Kimberly A. Garrett, Pooja S. Tandon

**Contents**

Table S1. Characteristics of groups observed using SOOPEN during recess, expressed as both counts and percentages.

Table S2. Relative difference in prevalence of MVPA over time at control and intervention schoolyards.

Table S3. Community use of control and intervention schoolyards over time.

**Table S1.**  Characteristics of groups observed using SOOPEN during recess, expressed as both counts and percentages.

|  | **Intervention** | | | | **Control** | | | |
| --- | --- | --- | --- | --- | --- | --- | --- | --- |
|  | **Pre** | | **Post** | | **Pre** | | **Post** | |
| **Number of groups** | N=676 | | N=490 | | N=157 | | N=156 | |
| **Activity level, n (%)** |  | |  | |  | |  | |
| Sedentary | 232 | (34%) | 80 | (16%) | 63 | (40%) | 49 | (31%) |
| Walking | 322 | (48%) | 298 | (61%) | 54 | (34%) | 73 | (47%) |
| Moderate/Vigorous | 119 | (18%) | 112 | (23%) | 40 | (26%) | 34 | (22%) |
| **Social interactions, n (%)** |  |  |  |  |  |  |  |  |
| Prosocial – physical | 139 | (21%) | 114 | (23%) | 44 | (28%) | 42 | (27%) |
| Prosocial – verbal | 232 | (34%) | 184 | (38%) | 56 | (36%) | 71 | (46%) |
| Neutral | 282 | (42%) | 191 | (39%) | 53 | (34%) | 43 | (28%) |
| Physical conflict | 10 | (2%) | 0 | (0%) | 2 | (1%) | 0 | (0%) |
| Verbal conflict | 5 | (1%) | 1 | (<1%) | 2 | (1%) | 0 | (0%) |
| **Perceived gender, n (%)** |  |  |  |  |  |  |  |  |
| Girls only | 289 | (43%) | 203 | (41%) | 60 | (38%) | 68 | (44%) |
| Boys only | 226 | (34%) | 177 | (36%) | 55 | (35%) | 62 | (40%) |
| Mixed group | 156 | (23%) | 107 | (22%) | 42 | (27%) | 26 | (17%) |
| **Group size, n (%)** |  |  |  |  |  |  |  |  |
| Alone | 270 | (40%) | 184 | (38%) | 47 | (30%) | 45 | (29%) |
| Small | 342 | (51%) | 249 | (51%) | 78 | (50%) | 92 | (59%) |
| Medium | 46 | (7%) | 47 | (10%) | 22 | (14%) | 16 | (10%) |
| Large | 18 | (3%) | 10 | (2%) | 10 | (6%) | 3 | (2%) |
| **Group location, n (%)** |  |  |  |  |  |  |  |  |
| Field/grass | 226 | (33%) | 144 | (29%) | 25 | (16%) | 41 | (26%) |
| Paved | 238 | (35%) | 165 | (34%) | 66 | (42%) | 59 | (38%) |
| Play structure | 212 | (31%) | 134 | (27%) | 66 | (42%) | 56 | (36%) |
| Nature area | - |  | 47 | (10%) | - |  | - |  |

**Table S2.** Relative change in prevalence of MVPA over time at control and intervention schoolyards.

|  | **Control school** | | **Intervention schools** | |
| --- | --- | --- | --- | --- |
|  | Relative change from pre to post (95% CI) | p-value | Relative change from pre to post (95% CI) | p-value |
| Full sample | 0.86 (0.57, 1.28) | 0.503 | 1.30 (1.04, 1.64) | 0.029 |
| Groups of girls | 0.76 (0.38, 1.51) | 0.430 | 1.38 (0.95, 2.00) | 0.109 |
| Groups of boys | 0.78 (0.43, 1.42) | 0.421 | 1.68 (1.10, 2.54) | 0.016 |
| Mixed-gender groups | 1.26 (0.53, 3.00) | 0.608 | 0.92 (0.60, 1.42) | 0.784 |

Table S2 includes risk ratios and 95% confidence intervals from Poisson regression analyses at the control school and at intervention schools, separately. Effect estimates reflect the relative change in MVPA over time (from pre to post). At intervention schools, we found a 30% increase (95% CI: 4%, 64%) in the probability of a group in the schoolyard engaging in MVPA over time from pre- to post-renovation. In contrast, we found that MVPA decreased over time (14% decrease, 95% CI: 43% decrease, 28% increase) at the control school.

**Table S3.** Community use of control and intervention schoolyards over time.

|  | **Control school** | | **Intervention schools** | |
| --- | --- | --- | --- | --- |
|  | Absolute change from pre to post (95% CI) | p-value | Absolute change from pre to post (95% CI) | p-value |
| All groups | -0.75 (-1.77, 0.27) | 0.149 | 0.18 (-0.07, 0.43) | 0.163 |
| Child-only groups | -0.67 (-1.39, 0.06) | 0.071 | 0.08 (-0. 70, 0.23) | 0.290 |
| Multi-generational groups | 0.04 (-0.17, 0.25) | 0.697 | 0.01 (-0.07, 0.10) | 0.756 |
|  | Relative change from pre to post  (95% CI) | p-value | Relative change from pre to post  (95% CI) | p-value |
| All groups | 0.45 (0.19, 1.10) | 0.082 | 1.48 (0.87, 2.52) | 0.153 |
| Child-only groups | 0.26 (0.07, 0.88) | 0.030 | 1.48 (0.72, 3.02) | 0.287 |
| Multi-generational groups | 1.22 (0.45, 3.30) | 0.692 | 1.10 (0.59, 2.08) | 0.757 |

Table S3 shows estimates of differences in community use of schoolyards over time—from pre to post—at the control school and intervention schools, separately. Changes on the absolute scale were estimated using linear regression and changes on the relative scale were estimated using Poisson regression. A trend of decreases over time, particularly for child-only groups, was observed at the control school. Effect estimates were generally in the opposite direction, corresponding to increases in community use from pre to post, at the intervention school.
